# *Burkholderia pseudomallei* rubrerythrin binds metals promiscuously in a pre-formed four-helix bundle

**DOI:** 10.1063/4.0001165

**Published:** 2025-10-27

**Authors:** Gabrielle R. Budziszewski, Miranda L. Lynch, M. Elizabeth Snell, Diana C. F. Monteiro, Sarah E. J. Bowman

**Affiliations:** 1 University at Buffalo-Hauptman Woodward Research Institute, Buffalo, NY, USA; 2 University at Buffalo, Jacobs School of Medicine, Department of Biochemistry, Buffalo, NY, USA; 3 University at Buffalo, Jacobs School of Medicine, Department of Structural Biology, Buffalo, NY, USA; 4 Center for Structural Biology, Center for Cancer Research, National Cancer Institute, National Institutes of Health, Frederick, MD, USA

## Abstract

In many bacteria, rubrerythrins are Ferritin-like superfamily proteins that participate in the oxidative stress response as radical scavengers. Burkholderia pseudomallei are a bacterial species that can cause the human disease meliodosis, a serious bacterial infection that requires an arduous course of antibiotics to clear. Formerly confined to the tropics, meliodosis cases have been reported in recent years in the southeastern United States as a result of altered climate circumstances. Rubrerythrin (Rbr) in B. pseudomallei is expressed under oxidative stress conditions, likely contributing to the ability of the organism to survive in soil environments and withstand the onslaught of the human immune system. Here, we characterize BpRbr as a promiscuous metal-binding protein which stably interacts with redox-capable first row transition metals manganese, cobalt, and iron. We confirm the presence of single metal species with the use of in-crystallo energy dispersive spectroscopy (EDX). Analysis of a metal-nonbinding variant of BpRbr reveals that the four-helix bundle structure of BpRbr is pre-formed, reconciling our observation that all metal binding sites are only partially occupied. Further work will explore the enzymatic activity of BpRbr, as other rubrerythrins have been observed to perform catalase, peroxidase, pyrophosphatase and superoxide dismutase functions. We anticipate that defining the mechanism of BpRbr and its required di-metal cofactor will enable therapeutic approaches to bacterial infections that target oxidative response pathways.

